# Interdisciplinary Orthopedic Management of Pediatric Patella Dislocation and Instability: An Educational Case Series

**DOI:** 10.7759/cureus.42860

**Published:** 2023-08-02

**Authors:** Anthony N Baumann, Sunita R Mengers, Anne M Dumaine, Morgan B Weber, R. Justin Mistovich

**Affiliations:** 1 Rehabilitation Services, University Hospitals Cleveland Medical Center, Cleveland, USA; 2 Orthopaedic Surgery, University Hospitals Cleveland Medical Center, Cleveland, USA; 3 Orthopaedic Surgery, Mayo Clinic, Rochester, USA

**Keywords:** physical therapy, orthopedic surgery, medial patellofemoral ligament, patellar instability, interdisciplinary

## Abstract

Pediatric patellar instability and/or dislocation is a challenging diagnosis category that requires an interdisciplinary team consisting of orthopedic surgeons and physical therapists for optimized patient outcomes. This educational case series outlines core concepts for three patients with unique patellar dislocation presentations. Case 1 is a 16-year-old male who presented with a history of five knee traumatic patellar dislocations with self-reduction and underwent knee arthroscopic surgery with debridement and microfracture of the patella chondral defect, arthroscopic lateral release to improve the patellar tilt, and medial patellofemoral ligament (MPFL) reconstruction. Case 2 is a 15-year-old female who presented with chronic knee pain and patella instability who underwent knee arthroscopic surgery with abrasion arthroplasty, microfracture of the patella, lateral release, tibial tubercle osteotomy medializing osteotomy, and MPFL reconstruction. Case 3 is a 14-year-old male who presented after a single episode of lateral patella dislocation and underwent open reduction and fixation of the lateral femoral condyle osteochondral fracture, a Grammont patellar medialization procedure, and MPFL reconstruction. All three patients received postoperative physical therapy (PT) to improve function and outcomes. These cases represent important concepts of patellar containment, risk factors for recurrent instability, associated pathology, and appropriate surgical care and postoperative rehabilitation. Furthermore, this case series highlights management decisions and pathways for three patients with different symptoms related to patellar instability, subsequent surgical correction, and postoperative physical therapy. Overall, interdisciplinary care of common pediatric orthopedic conditions can help improve patient outcomes and satisfaction. By understanding the biomechanics and decision-making surgical parameters regarding patellofemoral instability, clinicians can provide patients with better care.

## Introduction

Pediatric patella dislocations and subsequent instability are common injuries in children and adolescents, with an annual incidence of 43 cases per 100,000 patients, double than that of the adult population [1-3]. Patellar instability can result from a wide variety of activities as well as associated anatomic static and dynamic factors. Additionally, people who are young, have open physes, family history of patellar instability, or are women at a greater risk of patellar instability. In terms of functional knee anatomy, the stability of the patellofemoral joint is impacted by a combination of static and dynamic restraints, such as the extensor retinaculum and quadriceps muscle, in the surrounding osseous and soft tissue structures that can be addressed by orthopedic surgery and/or physical therapy (PT) [1-3]. An abnormality in or damage to any of these static or dynamic structures could increase risk of patellar dislocation and instability in the future, thus complicating treatment [2].

Almost half of pediatric patellar dislocations occur during physical activity [4]. Further re-injury is common as recurrent patellar dislocations can occur in up to 30-72% of all patients after their first patella dislocation [3]. Furthermore, injuries such as medial patellofemoral ligament (MPFL) rupture and osteochondral fractures can occur with traumatic patellar dislocation, further complicating patient care [2]. Given the various anatomic variables adding complexity, the potential for associated injuries, and the subsequent different treatment approaches, it is important for the entire interdisciplinary team to have a sound anatomic and clinical foundation so that shared treatment goals can be attained [5]. The purpose of this case series is to present three different patient presentations to model the complexity of pediatric patella dislocations along with subsequent surgical intervention and postoperative rehabilitation to allow for improved interdisciplinary education and collaboration.

## Case presentation

Case 1

**Figure 1:**
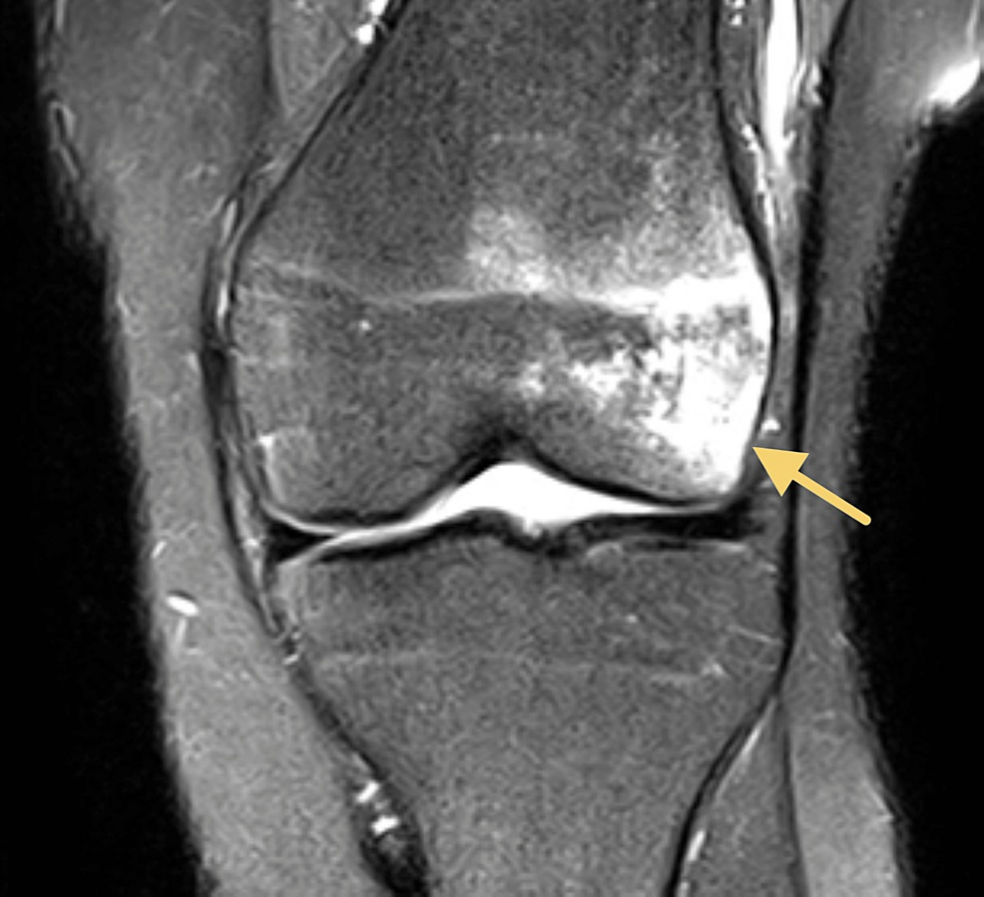
MRI for the first patient, revealing a bony contusion of the lateral femoral condyle The arrow indicates the bony contusion of the lateral femoral condyle.

**Figure 2:**
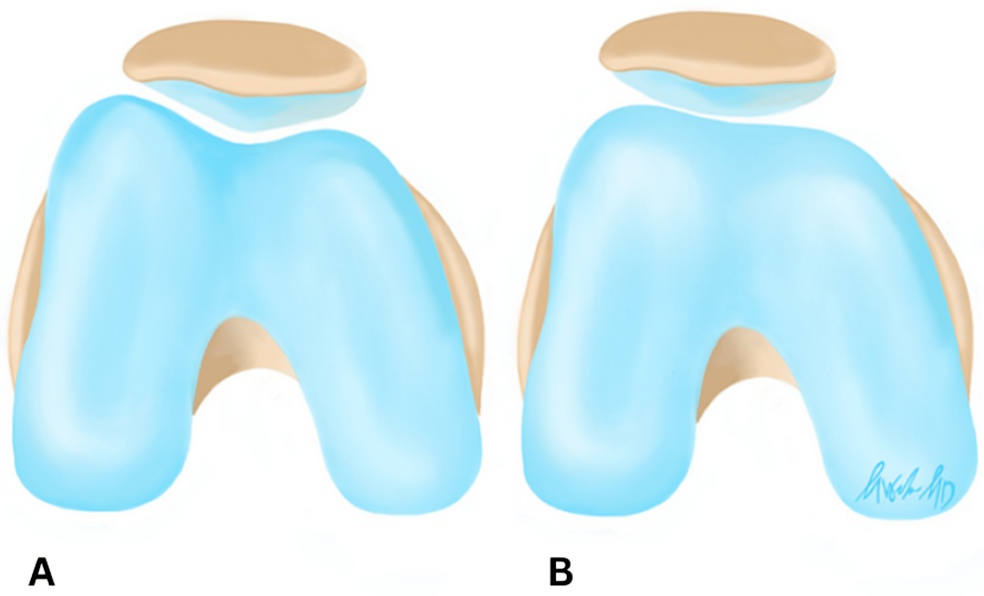
(A) A deep sulcus results in improved containment of the patella within the trochlea to decrease risk of patella dislocation; (B) a shallow sulcus provides an insufficient barrier to patella translation and increases the risk of patella dislocation. Image Credits: Morgan B. Weber

The patient is a 16-year-old male who presented to the orthopedic surgeon with a history of five left knee traumatic patellar dislocations with self-reduction without other significant past medical history (PMH). His physical exam demonstrated a moderate knee effusion, limited left knee range of motion (ROM), and a positive patellar apprehension test with no firm endpoint on lateral patellar translation. His contralateral exam revealed a normal, firm endpoint to lateral patellar translation. Based on the patient’s presentation, the differential diagnosis included patellofemoral pain, MPFL rupture, meniscus tear, osteochondral defect, and patellar instability. Given his history and physical exam findings, MRI was ordered to assess the three-dimensional alignment of his patellofemoral interface as well as identify any potential concomitant injury. MRI revealed osteochondral impaction injuries (i.e., bony contusions) of the lateral femoral condyle, an injury classically associated with acute patellar dislocations (Figure 1). His patella was well contained within his shallow groove and his extensor mechanism was not lateralized, though his patella did have some lateral tilt. Figure 2 demonstrates a graphic depicting an example of a shallow trochlear groove for improved visualization of this pathology. 

Given his recurrent instability, the patient underwent outpatient left knee arthroscopic surgery with debridement and microfracture of the patella chondral defect, arthroscopic lateral release to improve the patellar tilt, and MPFL reconstruction with a gracilis allograft. The specific technique for the MPFL reconstruction with a gracilis allograft has been described elsewhere in the literature [6]. Post-operative restrictions included toe-touch weight bearing (TTWB) for two weeks with progressive return to weight bearing as tolerated (WBAT). His knee ROM was initially limited from 0 to 30 degrees via a brace with the goal to increase his flexion by 10 degrees daily postoperatively. After his first postoperative appointment about one week after surgery, the patient was referred to PT to assist with return to prior level of function for sport and recreational tasks. The patient underwent several sessions of PT with a focus on appropriate exercises, education, and patient empowerment to promote independent management of symptoms and return to prior level of function.

Upon presentation to PT, this patient had no left knee pain or instability and was WBAT with bilateral axillary crutches. The patient had minimal left patella effusion with nearly full left knee ROM. Strength testing via manual muscle testing (MMT) was 5/5, but had difficulty performing functional tasks such as squatting and single-leg squat testing. The focus of the initial PT session was education on the anatomical and biomechanical influences impacting his knee symptoms, such as a shallow trochlear groove, as well as education on importance of compliance with his home exercise program (HEP). Plan of care was established for one time per week. Exercises at the first PT session included lower extremity stretches to address soft tissue restrictions (hamstring and gastrocnemius stretch), double leg squats, single leg squats and deadlifts, walking lunges, and resisted lateral stepping with limited ROM as needed. The patient only needed minimal cuing to use proper form during single-leg tasks. Jumping exercises were performed with the patient demonstrating decreased left lower extremity weight bearing at first. After cuing, patient was able to correct and bear weight equally on both lower extremities. At the following session, the patient performed some strengthening exercises (side lunges, forward step ups, lateral tap downs); however, much of the session focused on agility and plyometric exercises. Patient performed skipping, side shuffles, jogging short distances, and side to side jumps (modified speed skaters) to increase confidence and stability of his left knee. At this time, the patient was denying pain with any activity and only demonstrate slight difficulty with high level agility exercises. At the final discharge session, patient demonstrated full left knee ROM and satisfactory completion of various plyometric and agility exercises without pain. All exercises were able to be completed with proper form and patient denied any further concerns or unmet goals. The patient was then discharged successfully.

Case 2

**Figure 3:**
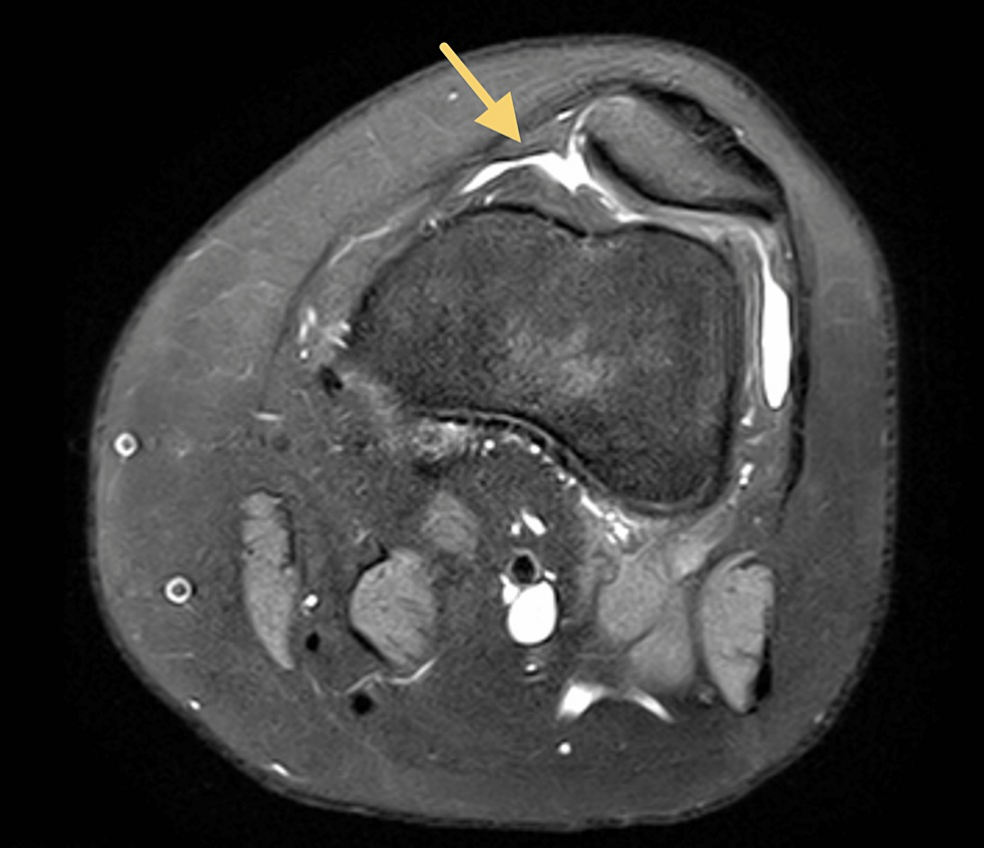
MRI of the knee for the second patient, which demonstrates trochlear dysplasia with a shallow groove, patellar tilt, and lack of patellar containment with the patella overhanging the lateral facet of the trochlea. The arrow indicates the region of trochlear dysplasia with a shallow groove, patellar tilt, and lack of patellar containment.

This patient is a 15-year-old female who presented with chronic left knee pain and patella instability without other significant PMH. Patient is an active competitive athlete involved in multiple sports and she reported that her patella frequently “pops out of place” while swimming, walking, or playing other sports. She had previously tried rest and PT with no relief in symptoms. On initial examination, no knee joint effusion was appreciated. She had full knee extension and could flex to 140 degrees. She had a positive patellar grind test as well as patellar apprehension. She had no other instability on ligamentous exam. Differential diagnosis for this patient included patellar instability without MPFL tear, patellar instability with MPFL tear, osteochondral defect, and patellofemoral pain. MRI of the knee demonstrated a small amount of lateral patellar tilt and lack of patellar containment within the trochlea, chondral loss on the medial facet of the patella as well as a lateralized tibial tuberosity. Figure 3 demonstrates patient’s MRI at initial presentation.

**Figure 4:**
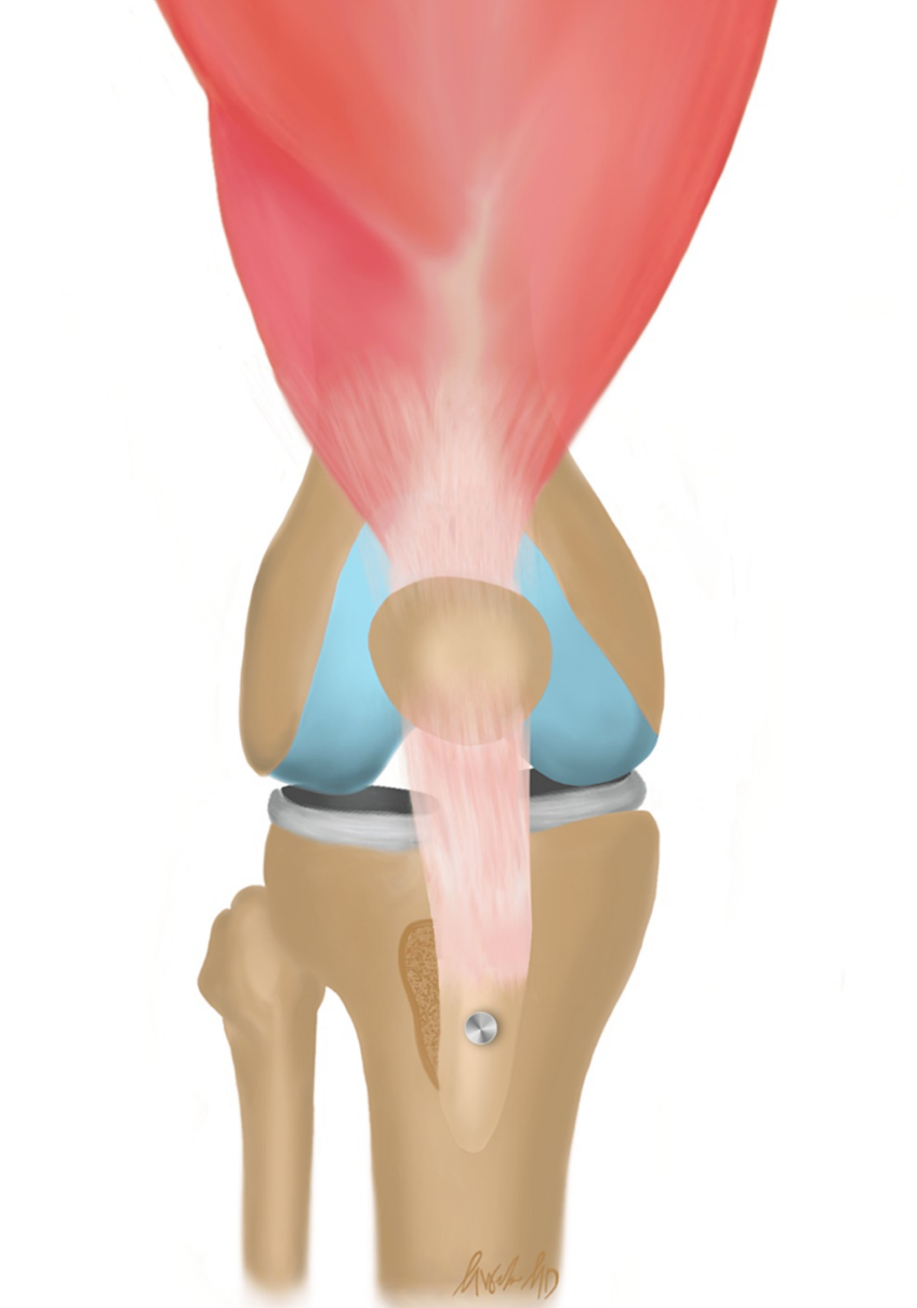
Tibial tubercle osteotomy and medialization of the tibial tubercle allows the patella to sit more centrally within the trochlea and provides tension to lateral translation. Image Credits: Morgan B. Weber

**Figure 5:**
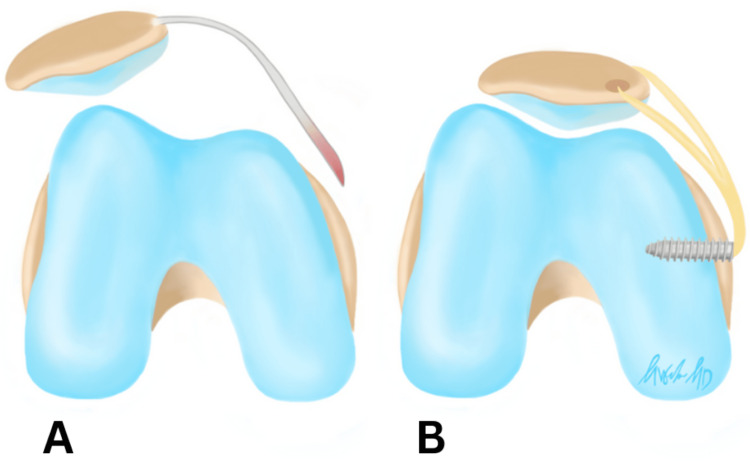
(A) An attenuated native MPFL results in lack of adequate resistance to lateral patella translation, which places patients at risk of lateral patella dislocation; (B) MPFL reconstruction results in improved containment of the patella within the trochlear groove and resistance to lateral patella translation. Image Credits: Morgan B. Weber MPFL: Medial patellofemoral ligament

Given multiple episodes of recurrent knee instability as well as radiographic evidence of chondral damage, the patient underwent left arthroscopic knee surgery. Arthroscopy with abrasion arthroplasty and microfracture of the patella was performed to address her chondral lesion. An arthroscopic lateral release was performed to correct her patellar tilt. Tibial tubercle osteotomy medializing osteotomy with a mild amount of anteriorizing was performed to create anatomic patellar containment as well as decrease contact pressure on her already damaged patellofemoral joint. Figure 4 illustrates a graphic depicting tibial tubercle osteotomy in order to better visualize the surgical intervention used for this patient. Patient underwent an MPFL reconstruction with a gracilis allograft to restore physiologic lateral translation of the patella [6]. Figure 5 demonstrates a graphic depicting the knee anatomy before and after MPFL reconstruction. Following the operation, the patient was initially instructed to remain TTWB and then was gradually transitioned to full weight bearing (FWB) when able. She attended PT to focus on ROM, strengthening, and agility training to allow return to sport.

Patient presented to post-operative PT two weeks after surgery with mild left knee pain, a left knee brace locked in extension, and restrictions of non-weight bearing (NWB) orders on the left lower extremity. Upon examination, her left knee extension ROM was 0 degrees of full extension and her left knee flexion ROM was 25 degrees. One week later, the patient presented to PT with increased left knee flexion ROM of 57 degrees. The patient was TTWB on her left lower extremity at this time. Exercises in the clinic at the current session involved straight leg raise with brace, prone hip extension, and quad sets with instructions for HEP. After eight total sessions over the next two months, the patient had achieved 0-139 degrees of left knee ROM with normalized gait mechanics with left knee brace donned. Exercises performed at this time included air squats with mirror feedback, lateral step downs on 4-inch step, bridges, resisted clam shells with resistance band, and wall squats. The patient denied any pain at the end at PT discharge per subjective reporting and her function has significantly improved per patient report. She was discharged and would only follow up with her surgeon as needed.

Case 3

**Figure 6:**
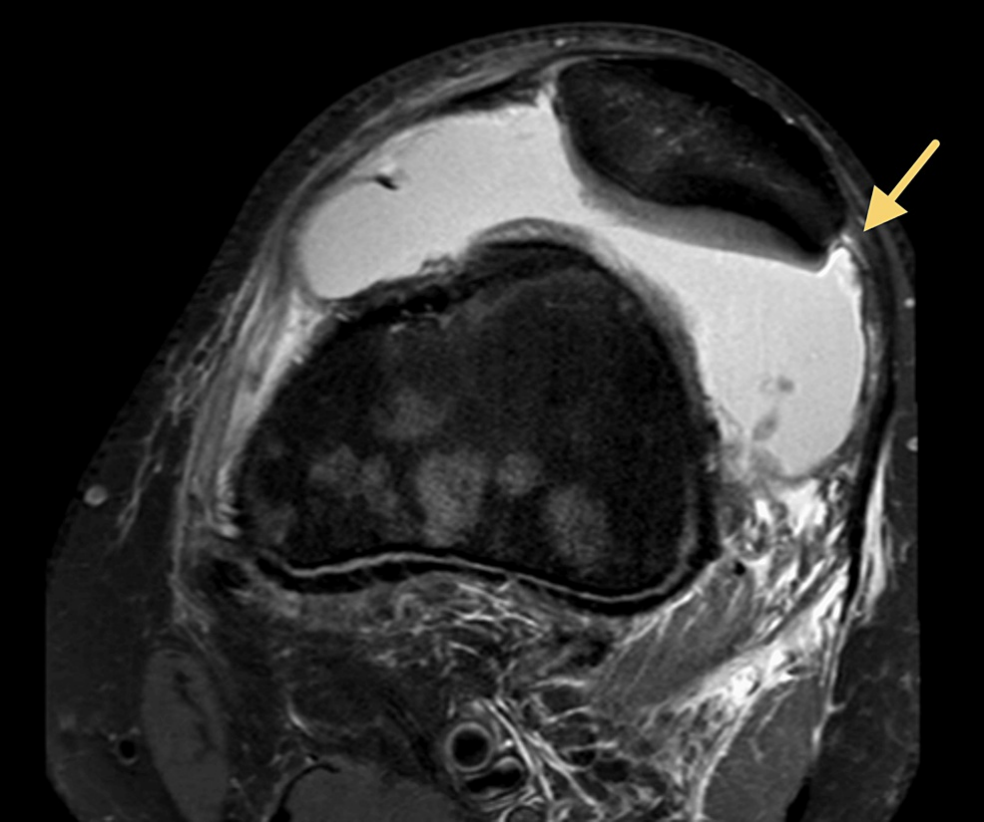
MRI of the knee of the third patient, demonstrating severe effusion, trochlear dysplasia, and severe lack of patellar containment. The arrow indicates the region of severe effusion, trochlear dysplasia, and the severe lack of patellar containment.

This patient is a 14-year-old male presenting after a single episode of left lateral patella dislocation that occurred while hiking without other significant PMH. Patient initially presented to the emergency room one day prior where a reduction was attempted with persistent patella lateralization secondary to a moderate effusion. On initial orthopedic evaluation, he was found to have tenderness over the lateral femoral condyle and a positive apprehension test as well as positive patella grind test. ROM was 0-40 degrees and limited by pain. Differential diagnosis for this patient was patellofemoral pain, patellar instability with MPFL tear, patellar instability without MFPL tear, fracture, and osteochondral defect. An MRI obtained demonstrated a large lipohemarthrosis with a persistently dislocated patella, a high-grade tear of the MPFL, multiple large osteochondral fragments, fracture of the lateral femoral condyle, and vertical fissuring of the patella. However, this growth plates remained open. Figure 6 depicts this patient’s MRI at initial presentation.

The patient subsequently underwent arthroscopic removal of loose bodies, microfracture of the patella, and lateral release. He also underwent open reduction and fixation of the lateral femoral condyle osteochondral fracture, a Grammont patellar medialization procedure, and MPFL reconstruction with a gracilis allograft [6]. Postoperatively, the patient was made NWB for four weeks with ROM limited to 0-30 degrees to allow the fracture to heal. He subsequently returned to the operating room for planned removal of screws at four weeks postoperatively, and was then made TTWB. After his postoperative visit after the second procedure, he began a PT program aimed to improve his strength and ROM.

The patient presented to PT two weeks after his second surgery with good progress, good healing, and minimal pain. He was wearing a locked brace throughout the day while ambulating, but unlocked the brace for passive motion. He also was wearing the brace at night, and was partial weight bearing with bilateral axillary crutches. Upon examination, his left knee extension ROM was lacking 8 degrees from full extension and his left knee flexion ROM was 98 degrees. Overall, his left knee strength was 3-/5 in extension and flexion, indicating partial ROM against gravity. The patient was unable to perform a left straight leg raise with significant quadriceps lag due to muscle inhibition. The prescribed HEP included knee extension stretches, heel slides, quads sets, and straight leg raise and the patient’s PT plan was two times per week at this time. At one-month after surgery, the patient denied any pain, continued to wear knee brace and use bilateral crutches, and still had mild difficulty with left straight leg raise. Upon examination, left knee extension ROM was 0 degrees of full extension and left knee flexion ROM was 110 degrees of flexion. Left knee extension and flexion strength was unchanged at this time; therefore, the patient received Russian neuromuscular electrical stimulation for quadriceps re-education and increased contraction. At two months after surgery, the patient denied any pain, was still wearing his brace, and still had significant limitations in left knee extension and flexion strength. His left knee extension ROM was 3 degrees past full extension and his left knee flexion ROM was 128 degrees. Exercises performed at this time in the clinic involved eccentric tap downs on a 6-inch step, single leg sit to stand without weight, lunges without weight, and resisted side steps. HEP was updated to include eccentric tap downs, squats, lunges, and side lying hip abduction. At three months after surgery, his knee extension and flexion strength were 3+/5, indicating minimal resistance against gravity, with only minimal quadriceps lag during straight leg raise. At this time, his exercises had progressed in the clinic to sports cord walking, single leg Romanian deadlifts, and single leg stance with ball tosses. At four months after surgery, patient’s left knee flexion and extension strength had improved to 5-/5 and he demonstrated full left knee ROM. At this time, exercises in the clinic involved dynamic, sport specific exercises such as ladder drills, cone touches, cutting drills, and jogging progressions. The patient continued to progress well and would return to sport in the near future. Finally, the patient was discharged with instructions to only follow-up if needed.

## Discussion

This case series helps to highlight the complexity and treatment options in the interdisciplinary management of patients with patellar dislocation and instability while explaining concepts related to surgical management and postoperative rehabilitation. This case series helps build on previous research in the field regarding interdisciplinary care for orthopedic conditions [1]. The current study helps the physical therapist to better understand patellar dislocations, be able to recognize possible concomitant pathologies leading to differing prognoses and surgical treatments, and improve interdisciplinary collaboration to improve patient outcomes.

A proper understanding of the passive and dynamic anatomical components of the knee relevant to patellar dislocations is crucial for interdisciplinary patient management. To begin, the patellar tendon, extensor retinaculum, bony anatomy, and MPFL provide passive stability to the patellofemoral joint [1,3]. Furthermore, it is important to note that the MPFL is the main passive restraint to patellar instability when the knee is extended, and the bony anatomy of the patellofemoral joint is the main passive restraint in greater angles of knee flexion [7]. When bony anatomy is inadequate, such as in trochlear dysplasia or a lateralized tibial tubercle, there can be increased risk of MPFL failure after surgery [2]. This factor requires increased vigilance and communication between the surgeon and the treating physical therapist.

The most important dynamic stabilizer of the patellofemoral joint is the quadriceps muscle [1]. The pull of the quadriceps muscle will attempt to pull the patella laterally; however, the vastus medialis oblique (VMO) and the MPFL help to resist this motion to keep the patella tracking correctly [1]. The overall positioning of the lower extremity, especially during sporting activities involving speed, can cause the quadriceps to exhibit more of a lateral force on the patella [3]. Therefore, it is no surprise that patellar dislocations almost always happen in the lateral direction [8]. Almost all patellar dislocations result in damage to the MPFL [4]. At times, if the MPFL is ruptured, the patella will track laterally even if other medial structures are still intact [1]. However, this will not hold true on every patient because of variations in anatomy. For example, patients with a deep trochlear groove and contained extensor mechanism could theoretically have a fully incompetent MPFL, yet still have minimal risk of recurrent instability due their individual knee architecture, and the preventative bony constraint of the lateral trochlear ridge and optimal dynamic extensor mechanism balance. Ideally, orthopedic surgeons and physical therapists will understand how all these passive and dynamic anatomic factors interact with one another in combination when planning surgery and rehabilitation [2].

In terms of initial patient management, plain radiographs and an MRI should be obtained in all first-time traumatic dislocations due to the incidence of chondral/osteochondral fractures that may not be seen with radiograph imaging [2]. MRI is additionally helpful in analyzing the patient’s knee for risk factors of recurrence, which can be used to tailor postoperative PT to reduce risk of further dislocations [5]. It is important to note which factors predispose a patient to surgical correction and to not delay referral to orthopedic surgery when appropriate.

Patellar instability can have different treatment pathways depending on multiple factors including presence of other concomitant injuries to knee, containment of the patella within the trochlea, and integrity of the MPFL. Primary patellar dislocations without other serious injuries and in patients with a well-contained patella may be treated non-operatively, thus requiring the physical therapist to have knowledge on how to treat as well as knowing when to refer to an orthopedic surgeon [4]. However, up to 25% of patients with a patellar dislocation have associated osteochondral fractures, requiring operative management [2]. Additionally, the recurrence rate after a first-time patellar dislocation ranges from 15-72%, indicating a potential for long-term impairment in function, with risk of further chondral injury with subsequent instability events [2,8]. Finally, long-term impairment is possible, as patients with a history of patellar dislocations and especially untreated recurrent instability have a higher incidence of developing patellofemoral arthritis [2]. When the quadriceps muscle doesn’t fully rehabilitate after an initial patellar dislocation, it is more likely that recurrent dislocations can occur [8]. For non-operative management of patellar dislocations, it is vital to take a complete patient history and perform a physical examination. Important questions include patient age, sex, type of activities, desired athletic goals, previous patellar dislocations (number of prior events, activities at the time of event, frequency of instability episodes), and imaging results. Physical exam should include gait mechanics, knee ROM, integrity of the MPFL via lateral glide testing, patellar mobility (in all directions), resting position of the patella (alta or baja), knee mechanics during step down task (valgus or normal alignment), quad strength, hip strengthening, calf tightness, and fear of movement or re-injury. Physical exam can also include the patella apprehension test, effusion grading, J sign, and hip internal and external ROM. Potential return to sport criteria includes full knee ROM, no swelling, strength that equals about 90% of the opposite side, and hop testing that equals about 90% of the opposite side. 

Clinicians need to discuss the risk of recurrence based on individual patient anatomy so that patients are informed their specific prognosis and optimal treatment pathways. If patients are not improving with conservative management, physical therapists should look for anatomical factors that could increase risk of recurrent patellar dislocations, which would indicate a need for surgical consultation [5,8].

Surgical intervention is necessary when a patella dislocation coincides with other pathologies, such as osteochondral fracture [7]. Likewise, if patellar instability continues after PT and other nonoperative interventions, surgery is indicated [7]. To broadly summarize operative goals, the surgeon should first ensure that the patella is contained within the trochlea. If the trochlea is dysplastic, even small amounts of lateralization of the extensor mechanism will lead to loss of patellar containment. In the first case, the patient was found to have a shallow trochlear groove as well as a compromised MPFL, though his patellar containment in that shallow groove was excellent. While a surgical procedure called trochleoplasty does exist to deepen the trochlear groove in patients with severe dysplasia, it is not without risks and limitations [9]. Several variations of the technique have been previously described including lateral facet elevation, sulcus deepening or subchondral deepening trochleoplasty, and recession wedge trochleoplasty [9,10]. Prior studies have demonstrated success in achieving stability, but residual pain and stiffness remain common concerns. Additional theoretical concerns include the fact that this new trochlear groove does not match the shape of the existing patella, and could potentially lead to osteoarthritis. Furthermore, should the trochleoplasty fail to heal, the articular cartilage about the trochlea is lost, a serious risk for which the only solution is total knee arthroplasty, which is unacceptable in a pediatric or adolescent patient. Therefore, this procedure is uncommonly performed in the United States and is typically reserved as a salvage procedure for severe dysplasia that has failed other treatments [9,11,12].

On the other hand, patients with a deeper trochlear groove can compensate for subtle extensor lateralization. If the patella is not contained, a procedure such as a tibial tubercle osteotomy to medialize the patella should be considered. Next, surgeons should address the stability of the patella to lateral translation. While a torn MPFL can heal (unlike, for example, an anterior cruciate ligament), it may now be too lax-or may have never been tight enough to begin with-to adequately protect the patella from lateral translation forces. In this case, a procedure such as MPFL reconstruction can be considered to restore physiologic lateral translation to the patella. Surgical options for MPFL reconstruction include autograft, allograft, and synthetic graft and involve the use of the gracilis, quadriceps, semitendinosus, adductor magnus, and tibialis anterior tendons [13-15]. The use of autografts vs. allografts in pediatric patients remains surgeon preference, though a recent retrospective review by Hendawi et al. suggests allografts confer greater survivorship amongst pediatric patients when compared to autografts [15,16]. Graft donor tissue is variable, though gracilis appears to be the most frequently utilized [15]. Additionally, any chondral injuries or other concomitant pathology that requires surgical intervention should be addressed.

As in the second patient case, the preoperative MRI can demonstrate focal chondral loss at the medial facet of the patella after patellar dislocation. Therefore, the patient in the second case presentation underwent abrasion arthroplasty in which a chondrotome was applied to the damaged articular surface of the patella to debride it back to a stable base that could form fibrocartilage. Additionally, a lateral retinacular release was performed to address findings of lateral patellar tilt. A tibial tubercle osteotomy was also performed to improve patellar containment. Anteriorization of the tibial tubercle can result in decreased contact forces at the patellofemoral joint while medialization of a lateralized tibial tubercle may improve congruity in patella tracking [17]. In a study evaluating the correlation between tibial tuberosity to trochlear groove (TT-TG) distance and patella instability in the pediatric population, the authors found a median TT-TG distance of 12.1 mm in patients with patella instability compared to 8.5mm in normal individuals (p< 0.001) [17,18]. The TT-TG distance was substantially elevated in our patient at 16 mm. However, TT-TG cannot account for all variables impacting the containment of the patella within the architecture of the trochlea [3]. As a result, new measurements have been proposed to directly quantify patellar containment as well as prognosticate the risk of recurrent instability in patients treated nonoperatively after a first-time dislocation. In a study by Weltsch et al, the authors proposed measuring the distance between the tibial tubercle and the lateral trochlear ridge [3]. They found that, unlike TT-TG, this measurement was able to independently predict recurrent instability, with patients with a tibial tuberosity-lateral trochlear ridge (TT-LTR) distance of >-1 mm (meaning that the tibial tubercle is at best barely-just 1-mm medial to the lateral trochlear ridge, and those with the tubercle equal to or lateral to the lip of the lateral trochlear ridge) having a 72% risk of recurrent dislocation. In the second patient case, the patient underwent medialization of the tibial tubercle to create patellar containment as well as slight anteriorization to reduce contact forces. 

As seen in the case of the third patient, concomitant pathologies such as osteochondral defect can occur along with pediatric patellar dislocations. During arthroscopic evaluation for patient three, the patient was found to have an osteochondral defect, necessitating removal of small loose bodies and fixation of the lateral femoral condyle as well as microfracture of the patella. In this case, the fixation of the large osteochondral fracture within the defect with two titanium headless compression screws provided sufficient coverage. However, in patients with failed fixation of an osteochondral fracture with a large chondral defect, a procedure such as Matrix Autologous Chondrocyte Implantation, which grows a patient’s cartilage cells in vitro for implantation, can be performed. Upon evaluation of the extensor mechanism, the quadriceps tendon tracked within the anterolateral third of the patella rather than in a neutral position, and the lateral structures were tight, requiring lateral retinaculum release similar to the procedure described in Case 2. Additionally, the tibial tubercle was substantially lateralized. However, because his physis about the proximal tibia remained open, a tibial tubercle bony osteotomy could not be performed. Therefore, a Grammont procedure was performed. Like the tibial tubercle osteotomy, the Grammont procedure aims to restore anatomical alignment in patients with a lateralized tibial tubercle [19]. However, unlike the tibial tubercle osteotomy, the Grammont involves carefully elevating the insertion of the patella tendon along with a periosteal sleeve from the tibial tubercle and translating and anchoring it medially to improve containment of the patella [19,20]. This patient also underwent MPFL reconstruction with a gracilis allograft for medial stabilization.

There are multiple limitations that impact the application and generalizability of this small case series to other patients. First, the small sample size doesn’t allow for in-depth discussion on the many different presentations of patellar instability; however, one of the purposes of this case series was to expound on the principles of interdisciplinary management for this condition, thus allowing for management of cases with differing presentations. Another limitation was the limited long-term follow-up with each patient, thus limiting solid recommendations aside from short term improvement. The long-term return to sport, return to functional activity, and level or lack of impairment into adulthood is not known for these pediatric patients. Therefore, more research is needed to determine how interdisciplinary care of pediatric patellar dislocations and instability impacts long term outcomes. This paper can be used to improve interdisciplinary collaboration and education as well as spur future research on this important topic.

## Conclusions

Pediatric patellar dislocation and instability is a challenging diagnosis that requires an interdisciplinary team consisting of orthopedic surgeons and physical therapists for optimized patient outcomes. This case series presents important concepts of patellar containment, risk factors for recurrent instability, associated pathology, and appropriate surgical care and postoperative rehabilitation for three different patient presentations. Furthermore, this case series highlights management decisions and pathways for patients with differing symptoms of patellar instability, subsequent surgical correction, and postoperative PT. Interdisciplinary care of common pediatric orthopedic conditions has the potential to improve patient outcomes and increase patient satisfaction.
